# Supplementary material for: Extracellular Vesicles in Young Serum Contribute to the Restoration of Age-Related Brain Transcriptomes and Cognition in Old Mice
Source: Int J Mol Sci. 2023 Aug 8;24(16):12550. doi: 10.3390/ijms241612550 (PMC10454174; doi:10.3390/ijms241612550)
Supplement: Supplementary file 1 [file ijms-24-12550-s001.zip › Suppl-Figures.pdf]

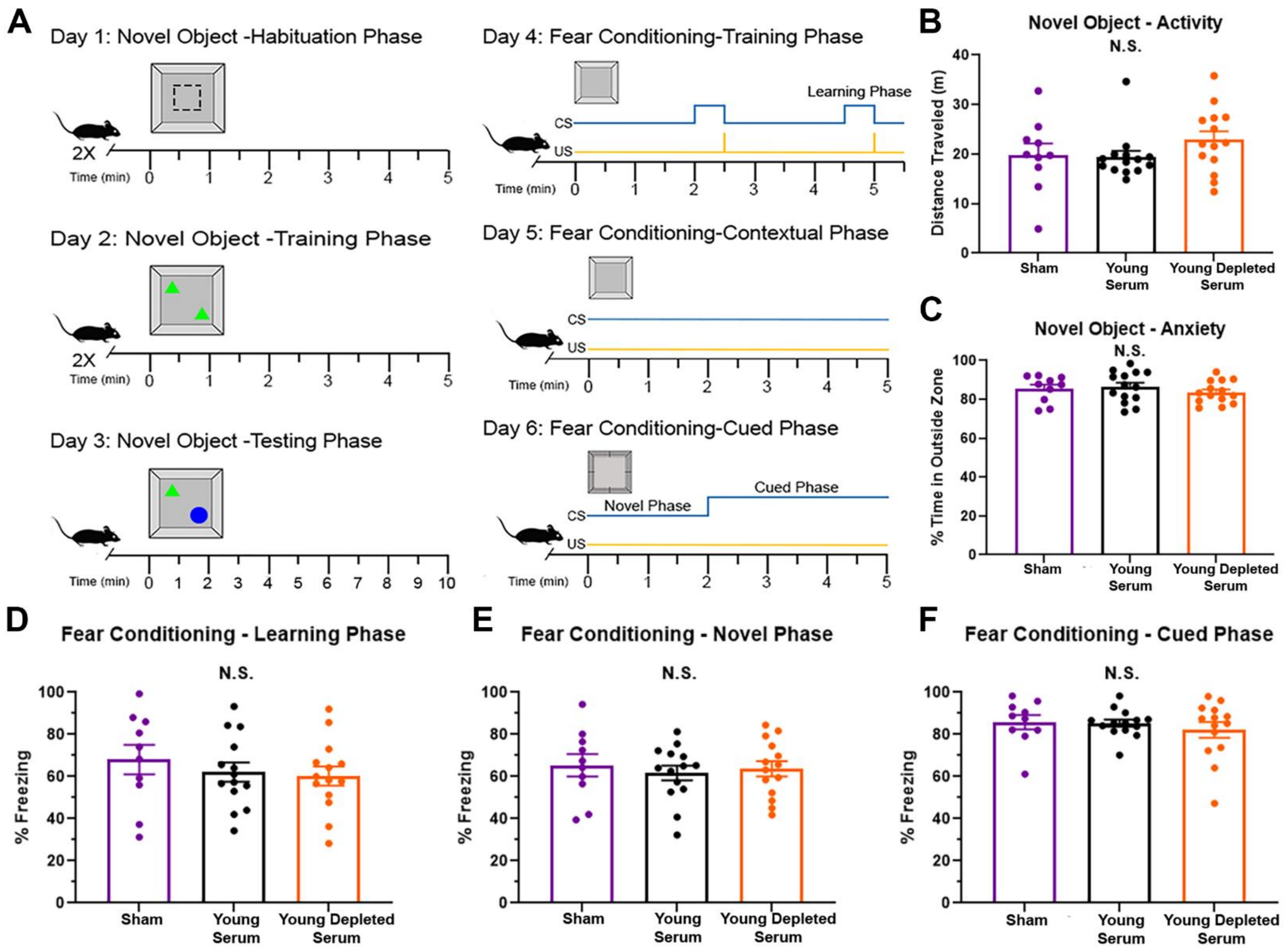

Figure S1. No significant effect on locomotor activity, anxiety like behavior, or learning as a result of young serum treatment. Behavioral testing schematic (**A**) depicting the different phases of novel object recognition and contextual cued fear condition over 6 days of training. There was no significant difference in the distance traveled (**B**) or percent time spent in the outside zone (**C**) during the open field habituation phase of novel objection recognition; indicating no change in locomotor activity or anxiety like behavior respectively. There was no significant difference in percent freezing during the learning (**D**), novel (**E**) or cued phase (**F**) for all experimental groups assessed during fear conditioning. These results indicate the observed changes in Figure 1 are due to cognitive function. Analysis by one-way ANOVA followed by Tukeys multiple comparison test. n=10 for sham; n=14 for young serum and young depleted serum with equal sex distribution. NS= no significance.

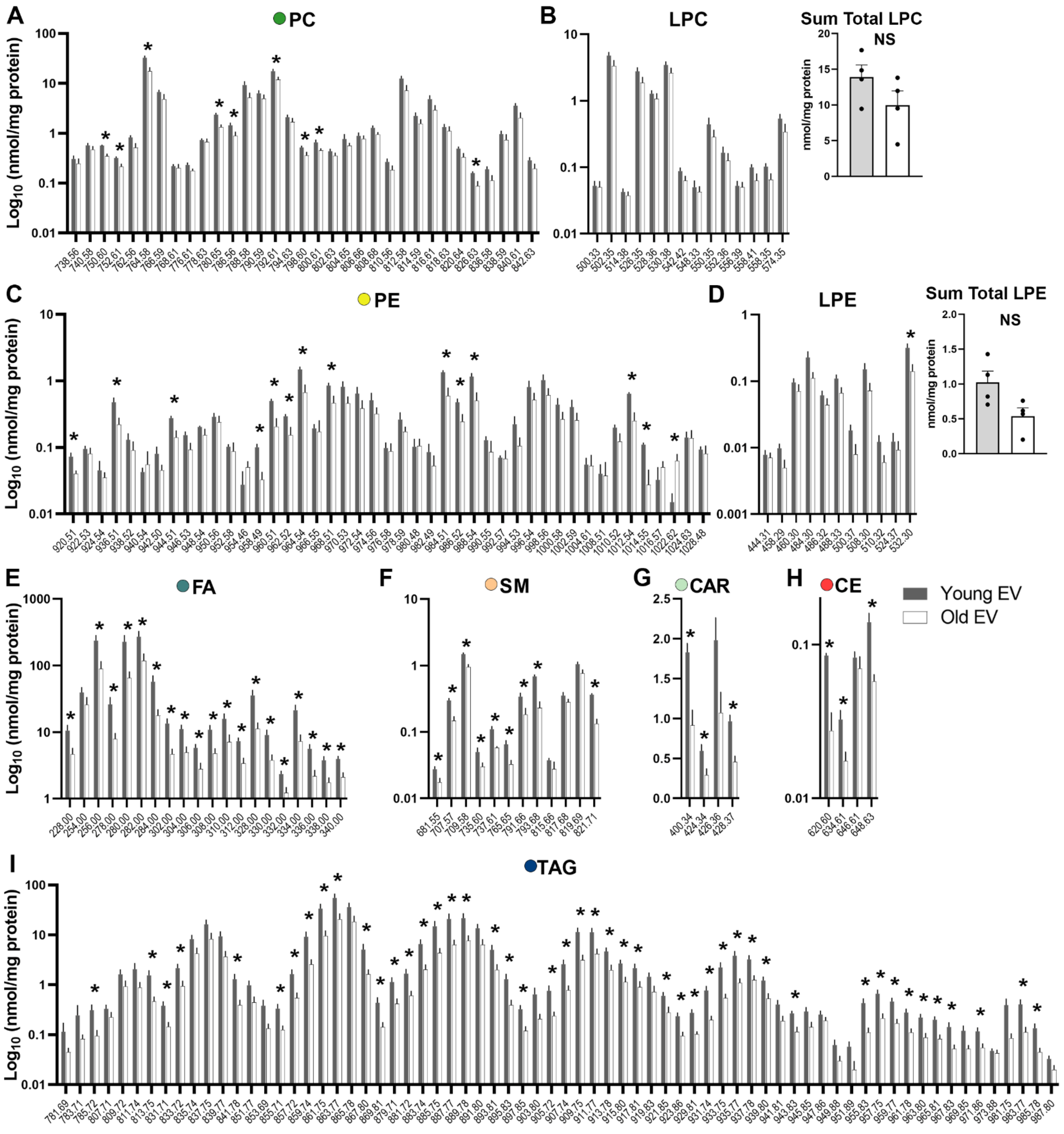

Figure S2. Serum EVs from aged animals are less lipidated with unique lipid species profiles when compared to serum EVs isolated from young animals. Lipid species-specific analysis determined using MDMS-SL of Phosphatidylcholine, PC (**A**); Lysophosphatidylcholine, LPC (**B**); Phosphatidylethanolamine, PE (**C**); Lysophosphatidylethanolamine, LPE (**D**); Fatty Acyl Chains in TAG, FA (**E**); Sphingomyelin, SM (**F**); Carnitine and Acetyl carnitine, CAR (**G**) Ceramide, CE (**H**), and Triacylglycerol, TAG (**I**) from serum EVs isolated from young and old mice. Analysis by two-tailed unpaired t-test. Bars represent mean ± SEM. n=4 for both young and old serum EVs with equal sex distribution. * $p < 0.05$, NS= no significance.
